# Oncogenic circDHTKD1 promotes tumor growth and metastasis of oral squamous cell carcinoma *in vitro* and *in vivo* via upregulating miR-326-mediated GAB1

**DOI:** 10.1590/1414-431X2020e10837

**Published:** 2021-07-16

**Authors:** Zhuangzhi Wu, Xiaoning He, Siqi Chen

**Affiliations:** 1 Department of Stomatology, The Second Affiliated Hospital of Hainan Medical University, Hainan, China

**Keywords:** circDHTKD1, Oral squamous cell carcinoma, miR-326, GAB1

## Abstract

Circular RNAs (circRNAs) have been extensively elucidated with regard to their significant implications in oral squamous cell carcinoma (OSCC). This study performed the functional investigation of circRNA dehydrogenase E1 and transketolase domain containing 1 (circDHTKD1) in OSCC. RNA expression levels of different molecules were measured via quantitative real-time polymerase chain reaction (qRT-PCR). Cellular behaviors were detected by 3-(4, 5-dimethylthiazol-2-y1)-2,5-diphenyl tetrazolium bromide (MTT) for cell viability, colony formation assay for clonal capacity, flow cytometry for cell apoptosis, wound healing assay for migration, and transwell assay for migration/invasion. Western blot was used for analyzing protein expression. RNA pull-down and dual-luciferase reporter assays were applied to assess the binding between targets. A xenograft tumor model was established in nude mice for *in vivo* experiments. Our expression analysis revealed that circDHTKD1 was upregulated in OSCC tissues and cells. circDHTKD1 knockdown was shown to impede OSCC cell growth and metastasis but motivate apoptosis. Additionally, circDHTKD1 served as a microRNA-326 (miR-326) sponge and the function of circDHTKD1 was achieved by sponging miR-326 in OSCC cells. Also, miR-326 inhibited OSCC development via targeting GRB2-associated-binding protein 1 (GAB1). circDHTKD1 could sponge miR-326 to alter GAB1 expression. Furthermore, circDHTKD1 contributed to OSCC progression *in vivo* via the miR-326/GAB1 axis. These data disclosed a specific circDHTKD1/miR-326/GAB1 signal axis in governing the malignant progression of OSCC, showing the considerable possibility of circDHTKD1 as a predictive and therapeutic target for clinical diagnosis and treatment of OSCC.

## Introduction

Oral cancer has become an alarming global matter as the 11th most common carcinoma and oral squamous cell carcinoma (OSCC) accounts for approximately 90% of cases of oral cancers (1). OSCC is closely associated with unhealthy lifestyle habits, such as smoking and excessive alcohol intake (2). The primary reason for mortality and relapse of OSCC is the regional or distant metastasis in many parts of the body, thus causing the poor prognosis for OSCC patients (3–5). Hence, it is quite necessary to untangle the tumorigenic mechanism for further target exploration.

Circular RNAs (circRNAs) and microRNAs (miRNAs) are two different sub-families of noncoding RNAs (ncRNAs) that have important regulation in the development of OSCC (6,7). Because of the covalently closed-loop structures by backsplicing, circRNAs are highly stable and more conserved than linear RNAs (8). circRNAs can function as the sponges of miRNAs to influence the interaction between miRNAs and 3′-untranslated regions (3′-UTRs) of target mRNAs to form the classical circRNA/miRNA/mRNA network in a variety of cancers (9–11).

Gao et al. (12) reported that circ-PKD2 impedes the carcinogenesis of OSCC via regulating the miR-204-3p/APC2 axis. Zhu et al. (13) reported that circRNA_100533 targets the miR-933/GNAS axis to inhibit the malignancy of OSCC. circPVT1 has been identified as a proliferation-promoting factor in OSCC via the regulation of STAT3 by sponging miR-125b (14). A previous study has indicated that circRNA dehydrogenase E1 and transketolase domain containing 1 (circDHTKD1) is upregulated in OSCC tissues (15), and our preliminary quantitative real-time polymerase chain reaction (qRT-PCR) detection also confirmed its upregulation in OSCC samples. However, its specific function in the progression of OSCC is still unclear. Tao et al. (16) reported that microRNA-326 (miR-326) is a tumor-inhibitory factor in OSCC by targeting MTA2. In addition, knockdown of GRB2-associated-binding protein 1 (GAB1) blocked cell proliferation, migration, and invasion in OSCC cells (17).

By making a hypothesis that circDHTKD1 can target miR-326 to regulate GAB1, we then designed the explorative experiments to prove it. The function and regulatory mechanism of circDHTKD1 in OSCC are two key points of this study, which may have an important meaning for the circDHTKD1 research in cancers.

## Material and Methods

### Patients and tissue specimens

OSCC patients (n=56) were subjected to surgical treatment at The Second Affiliated Hospital of Hainan Medical University, before which they had received no other therapies. Fifty-six paired OSCC tissues and matched normal adjacent tissues were collected after resection and then stored at -80°C for further experimental use. Written informed consent was obtained from all the involved patients. Biological principles of the Helsinki Declaration for research involving human samples were followed and this study was administered according to the ethical guidelines of the Ethical Committee of The Second Affiliated Hospital of Hainan Medical University.

### Cell culture

Human oral keratinocyte (HOK) cell line was purchased from QCHEN Bio (China) and OSCC cell lines (SCC9 and Cal27) were acquired from American Type Culture Collection (ATCC, USA). All cells were cultivated in a 37°C, 5% CO_2_ incubator with a culture solution of Dulbecco's modified Eagle's medium (DMEM; Gibco, USA), 10% fetal bovine serum (FBS; Gibco), and 1% penicillin/streptomycin antibiotics (Gibco).

### Cell transfection

Cells were transfected with small interfering RNA targeting circDHTKD1 (si-circDHTKD1) or short hairpin RNA against circDHTKD1 (sh-circDHTKD1, for stable transfection) following the specification of Lipofectamine™ 3000 Reagent (Invitrogen, USA). The miR-326 mimic and inhibitor (miR-326 and anti-miR-326) were used for the overexpression and inhibition of miR-326, and circDHTKD1 or GAB1 overexpression was achieved by the transfection of pCE-RB-Mam-circDHTKD1 (circDHTKD1) or pEXP-RB-Mam-GAB1 (GAB1) vector. All these oligonucleotides and vectors were bought from RIBOBIO (China), including their respective negative controls (si-NC, sh-NC, miR-NC, anti-miR-NC, pCE-RB-Mam, and pEXP-RB-Mam empty vectors).

### RNA acquisition and qRT-PCR

Total RNA was acquired via TRI Reagent (Sigma-Aldrich, USA) according to the manufacturer’s instructions. Reverse transcription was completed by RevertAid RT Reverse Transcription Kit (Thermo Fisher Scientific, USA) and expression was determined using RT-qPCR SYBR Green ROX Kit (Thermo Fisher Scientific). The miRNA quantification was performed by TaqMan™ MicroRNA Reverse Transcription Kit and TaqMan MicroRNA Detection Kit (Applied Biosystems, USA) following the manufacturer's instructions. The calculation of relative expression was carried out via the comparative cycle threshold (2^-ΔΔCt^) method. The internal gene for circRNA and mRNA was glyceraldehyde-phosphate dehydrogenase (GAPDH) and miRNA levels were normalized by U6. Primers for qRT-PCR were as follows: circDHTKD1, 5′-GACACCTACATCCCCCTGAA-3′ (forward) and 5′-TGAAGAGGGGGTTGATTTTG-3′ (reverse); DHTKD1, 5′-CTGCGGTTTGTTGGCATGAA-3′ (forward) and 5′-TCATCCGTCTCCTGGCAAAC-3′ (reverse); miR-326, 5′-GCCGAGCCTCTGGGCCCTTC-3′ (forward) and 5′-CAGTGCAGGGTCCGAGGTAT-3′ (reverse); miR-330-5p, 5′-TCGGCAGGTCTCTGGGCCTGTG-3′ (forward) and 5′-CGCAGGGTCCGAGGTAT-3′ (reverse); miR-338-3p, 5′-TGCGGTCCAGCATCAGTGAT-3′ (forward) and 5′-CCAGTGCAGGGTCCGAGGT-3′ (reverse); GAB1, 5′-TGGCAGCTCTTTACAAGCACC-3′ (forward) and 5′-TCATGAGCAACAGGTAGTCTTGA-3′ (reverse); GAPDH, 5′-TCAAGGCTGAGAACGGGAAG-3′ (forward) and 5′-TCGCCCCACTTGATTTTGGA-3′ (reverse); U6, 5′-CTCGCTTCGGCAGCACATATACTA-3′ (forward) and 5′-AAATATGGAACGCTTCACGA-3′ (reverse).

### Analysis of stability and localization

For stability analysis, 5 μg total RNA was incubated with 20 U Ribonuclease R (RNase R; Epicentre Technologies, USA) at 37°C for 1 h and qRT-PCR was carried out to assay circDHTKD1 and linear DHTKD1 levels. In addition, SCC9 and Cal27 cells were treated with 2 mg/mL actinomycin D (Sigma-Aldrich) for 0, 4, 8, 12, or 24 h followed by qRT-PCR detection for circDHTKD1 and linear DHTKD1. For the cellular localization, PARIS™ Kit (Invitrogen) was applied for isolating the nuclear or cytoplasmic RNA and circDHTKD1 was examined by qRT-PCR using GAPDH and U6 as the control groups.

### MTT assay

After seeding SCC9 and Cal27 cells (1×10^4^/well) onto 96-well plates for 24 h, transfection was conducted for 0, 12, 24, and 48 h. CyQUANT™ MTT Cell Viability Assay (Invitrogen) was used for the detection of cell viability. In brief, 10 μL MTT [3-(4,5-dimethylthiazol-2-y1)-2,5-diphenyl tetrazolium bromide] was instilled into each well for 4 h-incubation and the generated formazan was dissolved by adding 100 µL SDS-HCI solution. Finally, the absorbance was read under a microplate reader (Thermo Fisher Scientific) at 570 nm.

### Colony formation assay

After transfection for 24 h, cells were plated onto 6-well plates with the initial density of 500 cells/well and cultured in DMEM medium containing 10% FBS under the normal condition for 14 days. At room temperature, the colonies were immersed in methanol (Sigma-Aldrich) for 10-min fixation and in crystal violet (Sigma-Aldrich) for 15-min staining. Three biological and three technical replicates were performed for each group. The images of colonies were obtained by a light microscope (Thermo Fisher Scientific) and all colonies in each well were manually counted with 50 cells as one colony.

### Flow cytometry

Seventy-two hours after cell transfection, Annexin V-FITC/Propidium Iodide (PI) Apoptosis Detection Kit (Dojindo, Japan) was used for apoptosis analysis in strict accordance with the manufacturer’s instructions. Then, the flow cytometer (BD Biosciences, USA) was used to differentiate the apoptotic cells. Annexin V (+)/PI(-) stained cells were considered as the viable apoptotic cells while Annexin V(+)/PI(+) stained cells represented the apoptotic cells. Apoptosis rate was calculated using the ratio of apoptotic cells / total cells × 100%.

### Wound healing assay

Transfected SCC9 and Cal27 cells (2×10^5^) were inoculated onto 6-well plates to culture for monolayer confluence. Then, two straight scratches were created using a sterile 200 μL pipette tip, followed by cell washing with phosphate buffer solution (PBS) and incubation in cell medium without FBS. Images were obtained by a light microscope (40×, Thermo Fisher Scientific) at 0 and 24 h and the wound healing rate (%) is reported as wound width_(0 h)_ - wound width_(24 h)_ / wound width_(0 h)_.

### Transwell assay

Migration and invasion were analyzed using transwell chambers (Corning Inc., USA) and matrigel (Corning Inc.) pre-coated transwell chambers, respectively. Cells (1×10^5^) resuspended in serum-free medium were seeded into the upper chamber, while 10% FBS+DMEM medium was added into the lower chamber. Twenty-four hours later, cells across the membranes were collected and dyed using 4% paraformaldehyde and crystal violet (Sigma-Aldrich). Migrated and invaded cells were photographed under an inverted microscope (100× magnification, Olympus, Japan) and counted.

### Western blot

To obtain total proteins, cells were lysed in RIPA Buffer (Cell Signaling Technology (CST), USA). BCA Protein Assay Kit (CST) was employed for the determination of protein concentration. According to previous publications (18,19), western blot was carried out using 20 µg proteins in each group. Primary antibodies were matrix metalloproteinase 2 (MMP2, CST, #40994, 1:1000), MMP9 (CST, #13667, 1:1000), GAB1 (CST, #3232, 1:1000), and GAPDH (CST, #5174, 1:1000). Anti-rabbit IgG, HRP-linked antibody (CST, #7074, 1:2000) was used to combine with primary antibody to form the protein complex, followed by the detection by SignalFire™ Plus ECL Reagent (CST). Ultimately, the grey analysis of each gene was performed via the ImageLab software version 4.1 (Bio-Rad, USA) and GAPDH acted as the housekeeping gene for other objective genes.

### RNA pull-down assay with circDHTKD1 probe

C-1 magnetic beads (Life Technologies, USA) were pre-coated with circDHTKD1 probe or Oligo probe (RIBOBIO) at room temperature for 2 h. SCC9 and Cal27 cells transfected with vector or circDHTKD1 were lysed and incubated with the probe-coated magnetic beads at 4°C overnight and qRT-PCR was used for the expression analysis of circDHTKD1. In addition, cell lysates of SCC9 and Cal27 cells were also incubated with the above probe-coated magnetic beads followed by the enrichment detection of different miRNAs (miR-326, miR-330-5p, and miR-338-3p).

### Dual-luciferase reporter assay

circDHTKD1 sequence (wild-type, WT) or its mutated sequence (mutant-type, MUT), based on the miR-326 binding sites, was amplified to perform molecular cloning via the basic luciferase gene vector pGL3-control (Promega, USA). The luciferase vector containing circDHTKD1 WT or MUT sequence was termed as circDHTKD1 WT and circDHTKD1 MUT. GAB1 3′-UTR-WT and GAB1 3′-UTR-MUT were constructed in the same way. Each plasmid was then transfected into SCC9 and Cal27 cells together with miR-326 or miR-NC. After transfection for 48 h, the firefly and renilla luciferase signals were measured by the dual-luciferase reporter assay system (Promega), according to the manufacturer’s guidelines. The ratio of firefly and renilla was used to denote the relative luciferase activity.

### *In vivo* experiment

To establish the xenograft tumor model, six-week-old male BALB/c nude mice (Vital River Laboratory Animal Technology, China) were subcutaneously injected with SCC9 cells (2×10^6^/0.2 mL PBS) or sh-NC/sh-circDHTKD1 transfected SCC9 cells (6 mice for each group). After cell inoculation, tumor length (L) and width (W) were measured by a digital caliper every 5 days, followed by the calculation of tumor volume (L × W^2^ × 0.5). Twenty-five days later, those mice were sacrificed by displacing the 60% air of the cage using the flow rate of CO_2_. After the dissected tumors were weighed, the total RNA and protein were extracted for qRT-PCR and western blot analyses. Animal care followed the Management and Use Guidelines of Laboratory Animals of the National Institutes of Health (NIH) and this animal assay was approved by the Animal Ethical Committee of The Second Affiliated Hospital of Hainan Medical University.

### Statistical analysis

SPSS 24.0 (IBM Corp., USA) was used for statistical analysis, and figure production was completed using Graphpad Prism 7 (GraphPad Inc., USA). All data are reported as means±SD with three independent experiments of n=3. Student's *t*-test and one-way analysis of variance (ANOVA) followed by Tukey's test were adopted for analysis of different groups. P<0.05 was considered to be a significant difference.

## Results

### Identification of upregulated circDHTKD1 in OSCC tissues and cells

**Figure 1:**
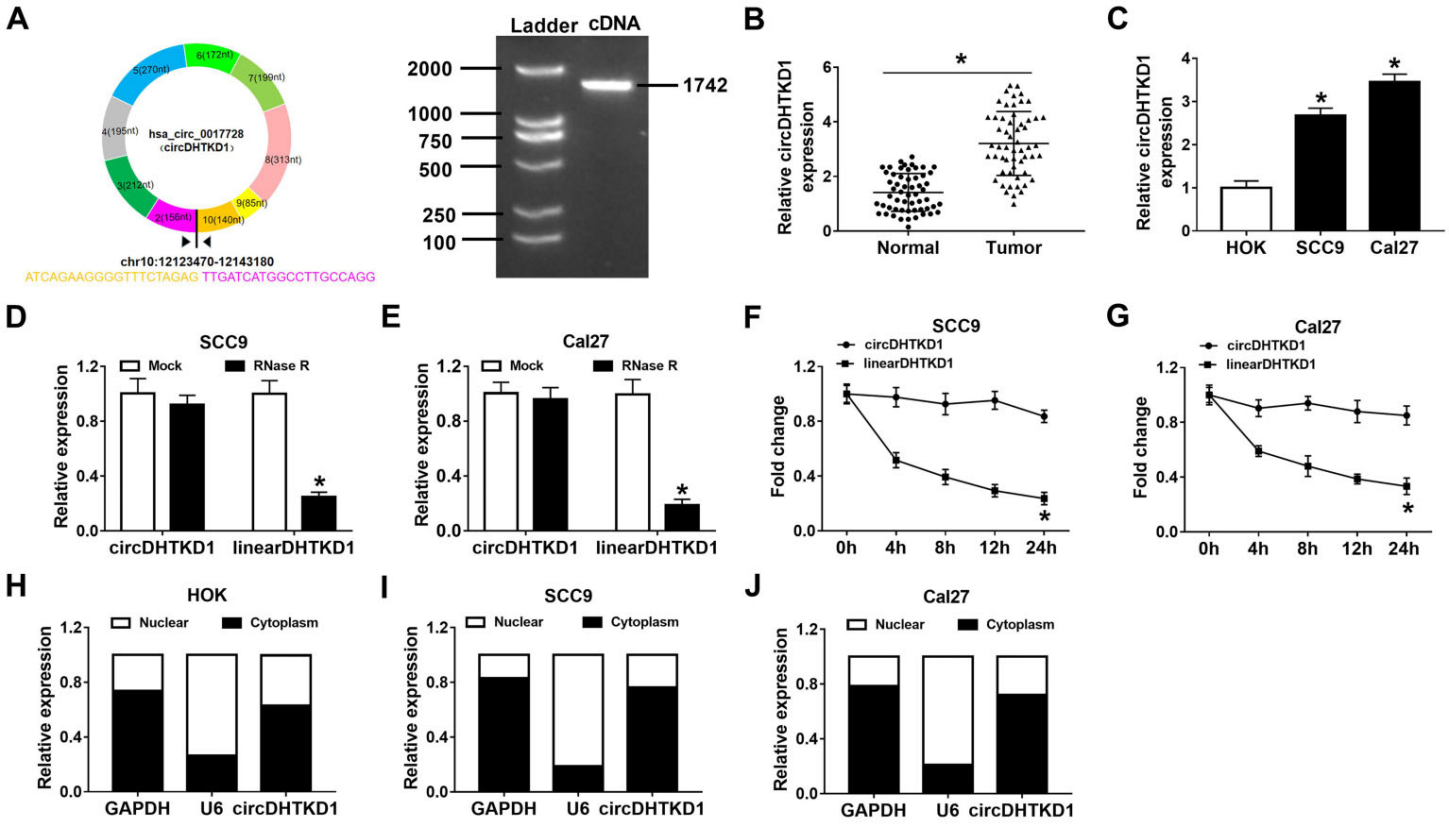
Identification of upregulated circDHTKD1 in oral squamous cell carcinoma (OSCC) tissues and cells. **A**, Genetic information of circDHTKD1 and the analysis of circDHTKD1 length by conventional PCR. **B** and **C**, qRT-PCR was applied for expression of circDHTKD1 in OSCC/normal tissues and OSCC (SCC9 and Cal27) cells/HOK cells. Three biological and three technical replicates were performed. circDHTKD1 and linear DHTKD1 levels were determined using qRT-PCR after RNase R treatment for total RNA (**D** and **E**) and actinomycin D treatment for OSCC cells (**F** and **G**). Three biological and three technical replicates were performed. **H-J**, The detection of circDHTKD1, GAPDH, and U6 was carried out via qRT-PCR in nuclear and cytoplasmic fractions of HOK cells and OSCC cells. Three biological and three technical replicates were performed. Data are reported as means±SD. *P<0.05 (Student's *t*-test).

circDHTKD1 originates from the exon 2-10 of DHTKD1 gene in the chr10: 12123470-12143180, and our conventional PCR confirmed that the length of circDHTKD1 was 1742 bp as shown in Figure 1A. Through the detection of circDHTKD1 in our collected 56 OSCC samples relative to normal samples, circDHTKD1 was identified to be overexpressed as a dysregulated circRNA (Figure 1B). The clinicopathological characteristics have demonstrated that high expression of circDHTKD1 was associated with the clinical stages and tumor size (Table 1). Also, circDHTKD1 expression was higher in SCC9 and Cal27 cells than in HOK cells (Figure 1C). No difference of circDHTKD1 level was observed but linear DHTKD1 was largely decreased after RNase R treatment, showing the high stability of circDHTKD1 as a circRNA (Figure 1D and E). Also, a time course with actinomycin D showed that the half-life of circDHTKD1 was longer than linear DHTKD1 (Figure 1F and G). The exonic circRNAs are commonly localized in the cytoplasm (20). Through comparing the expression of circDHTKD1 with the levels of cytoplasmic GAPDH and nuclear U6, we identified that circDHTKD1 was principally localized in the cytoplasm not only in normal HOK cells (Figure 1H) but also in SCC9 and Cal27 cells (Figure 1I and J). circDHTKD1 was identified as an upregulated circRNA in OSCC.

**Table 1:** Correlation between circDHTKD1 expression and clinicopathological characteristics in oral squamous cell carcinoma patients (n=56).

Clinicopathologic parameters	Patients	circDHTKD1 expression		P value
		Low (n=28)	High (n=28)	
Gender				0.2801
Male	32	18	14	
Female	24	10	14	
Age (years)				0.2646
≤60	36	20	16	
>60	20	8	12	
Clinical stage				0.0145^*^
I-II	23	16	7	
III-IV	33	12	21	
Tumor size (cm)				0.0533
<3	21	14	7	
≥3	35	14	21	
Differentiation				0.1582
Well/Moderate	37	21	16	
Poor	19	7	12	

P<0.05, chi-squared test.

### Silencing circDHTKD1 suppressed cell growth and metastasis and induced apoptosis of OSCC cells

**Figure 2:**
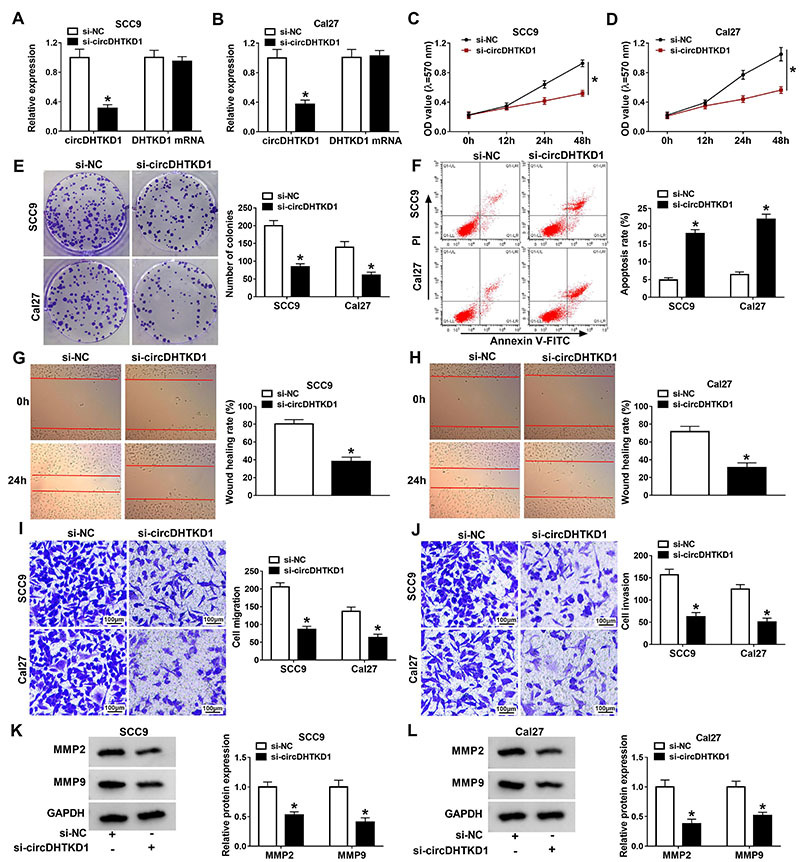
Silencing circDHTKD1 suppressed cell growth and metastasis and induced apoptosis of oral squamous cell carcinoma (OSCC) cells. SCC9 and Cal27 cells were transfected with si-NC (negative control) or si-circDHTKD1. **A** and **B**, The levels of circDHTKD1 and DHTKD1 mRNA were assayed by qRT-PCR. MTT and colony formation assays were, respectively, used for analyzing cell viability (**C** and **D**) and clonal ability (**E**). **F**, Apoptosis was measured by flow cytometry. **G** and **H**, Wound healing assay was carried out to assess cell migration ability. Transwell assay was used for analyzing cell migration (**I**) and invasion (**J**) (scale bar: 100 μm). **K** and **L**, The protein levels of MMP2 and MMP9 were detected using western blot. Three biological and three technical replicates were performed in each assay. Data are reported as means±SD. *P<0.05 (Student's *t*-test).

Transfection of si-circDHTKD1 was applied for expression knockdown of circDHTKD1 in SCC9 and Cal27 cells. As shown in Figure 2A and B, circDHTKD1 was significantly downregulated in the si-circDHTKD1 group compared with the si-NC group but DHTKD1 mRNA expression was unchanged. With the knockdown of circDHTKD1, cell viability (Figure 2C and D) and clonal ability (Figure 2E) were significantly inhibited while cell apoptosis rate was increased (Figure 2F) in SCC9 and Cal27 cells. By the wound healing assay, we found that cell migratory ability was restrained by the downregulated circDHTKD1 in SCC9 and Cal27 cells (Figure 2G-H). Also, cell migration (Figure 2I) and invasion (Figure 2J) by transwell assay were reduced after transfection of si-circDHTKD1 compared to si-NC transfection. MMPs can reflect cell invasion and aggressiveness in many tumors (21,22). Our western blot indicated the inhibitory effects of si-circDHTKD1 on MMP2 and MMP9 protein levels compared with the si-NC group (Figure 2K and L), indicating that circDHTKD1 downregulation blocked cell invasion. Taken together, silencing circDHTKD1 suppressed OSCC cell growth and metastasis but enhanced apoptosis.

### circDHTKD1 acted as a molecular sponge of miR-326

**Figure 3:**
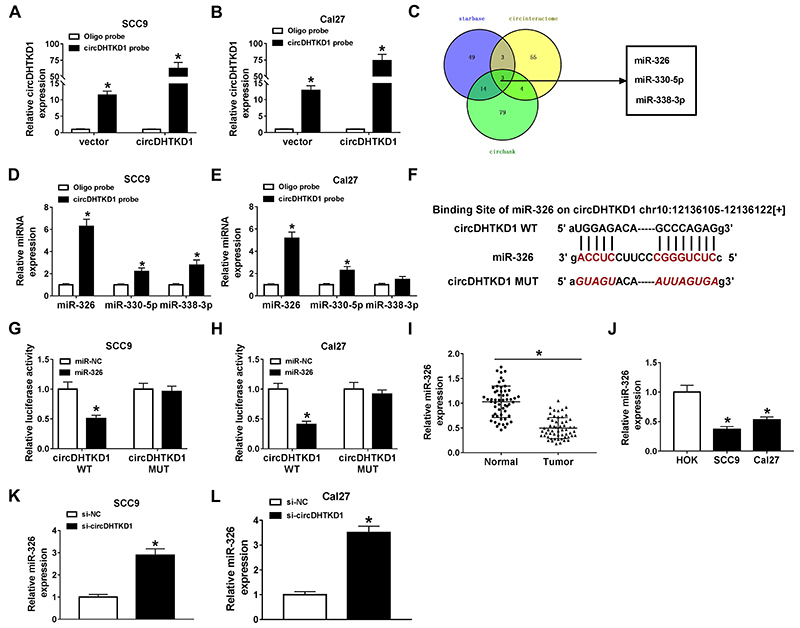
circDHTKD1 acted as a molecular sponge of miR-326. **A** and **B**, RNA pull-down assay was performed in SCC9 and Cal27 cells transfected with vector or circDHTKD1 to analyze the action of circDHTKD1 probe. **C**, The common miRNAs from starBase, circBank, and CircInteractome were analyzed by Venn diagram. **D** and **E**, Different miRNAs levels were examined by qRT-PCR after RNA pull-down with circDHTKD1 probe in SCC9 and Cal27 cells. **F**, The circDHTKD1 and miR-326 interaction was predicted by starBase. **G** and **H**, The binding of circDHTKD1 with miR-326 was identified by dual-luciferase reporter assay. qRT-PCR was employed to determine the relative expression of miR-326 in oral squamous cell carcinoma tissues (**I**) and cells (**J**). **K** and **L**, The effect of si-circDHTKD1 on miR-326 level was analyzed via qRT-PCR using si-NC (negative control) as the control group. Three biological and three technical replicates were performed in each assay. Data are reported as means±SD. *P<0.05 (Student's *t*-test and one-way ANOVA followed by Tukey's test).

To search for the miRNA targets of circDHTKD1, circDHTKD1 probe was designed and its functional affirmation was performed first. RNA pull-down assay showed that circDHTKD1 probe caught more circDHTKD1 in vector or circDHTKD1 transfection groups than Oligo probe, and circDHTKD1 expression was higher by circDHTKD1 probe following circDHTKD1 overexpression (Figure 3A and B), which suggested that circDHTKD1 probe was successful to capture circDHTKD1. Venn diagram was used to analyze the common miRNAs predicted by starBase (starBase (https://bio.tools), circBank (https://:circbank.cn), and CircInteractome (https://circinteractome.nia.nih.gov). As Figure 3C describes, three miRNAs (miR-326, miR-330-5p, and miR-338-3p) were selected as the potential candidate targets for circDHTKD1. Then, pull-down assay revealed that miR-326 was the most significant miRNA pulled down by circDHTKD1 probe compared with Oligo probe (Figure 3D and E), thus the target relation of miR-326 and circDHTKD1 was further confirmed. starBase showed that there were two target regions between miR-326 and circDHTKD1 sequences (Figure 3F). Dual-luciferase reporter assay showed that miR-326 could interact with circDHTKD1 to inhibit the luciferase signal of circDHTKD1 WT plasmid, with no effect on the mutated circDHTKD1 MUT plasmid (Figure 3G and H). OSCC tissue samples and cells (SCC9 and Cal27) were found to express miR-326 at a low level, relative to normal tissues and HOK cells (Figure 3I and J). In addition, miR-326 expression was elevated after circDHTKD1 knockdown in SCC9 and Cal27 cells (Figure 3K and L). The miR-326 expression was increased perhaps because some miR-326 absorbed by circDHTKD1 was released after circDHTKD1 was knocked-down. Thus, circDHTKD1 had the sponge effect on miR-326 in OSCC cells.

### Inhibition of miR-326 partly restored the cancer-inhibitory function of downregulated circDHTKD1 in OSCC cells

**Figure 4:**
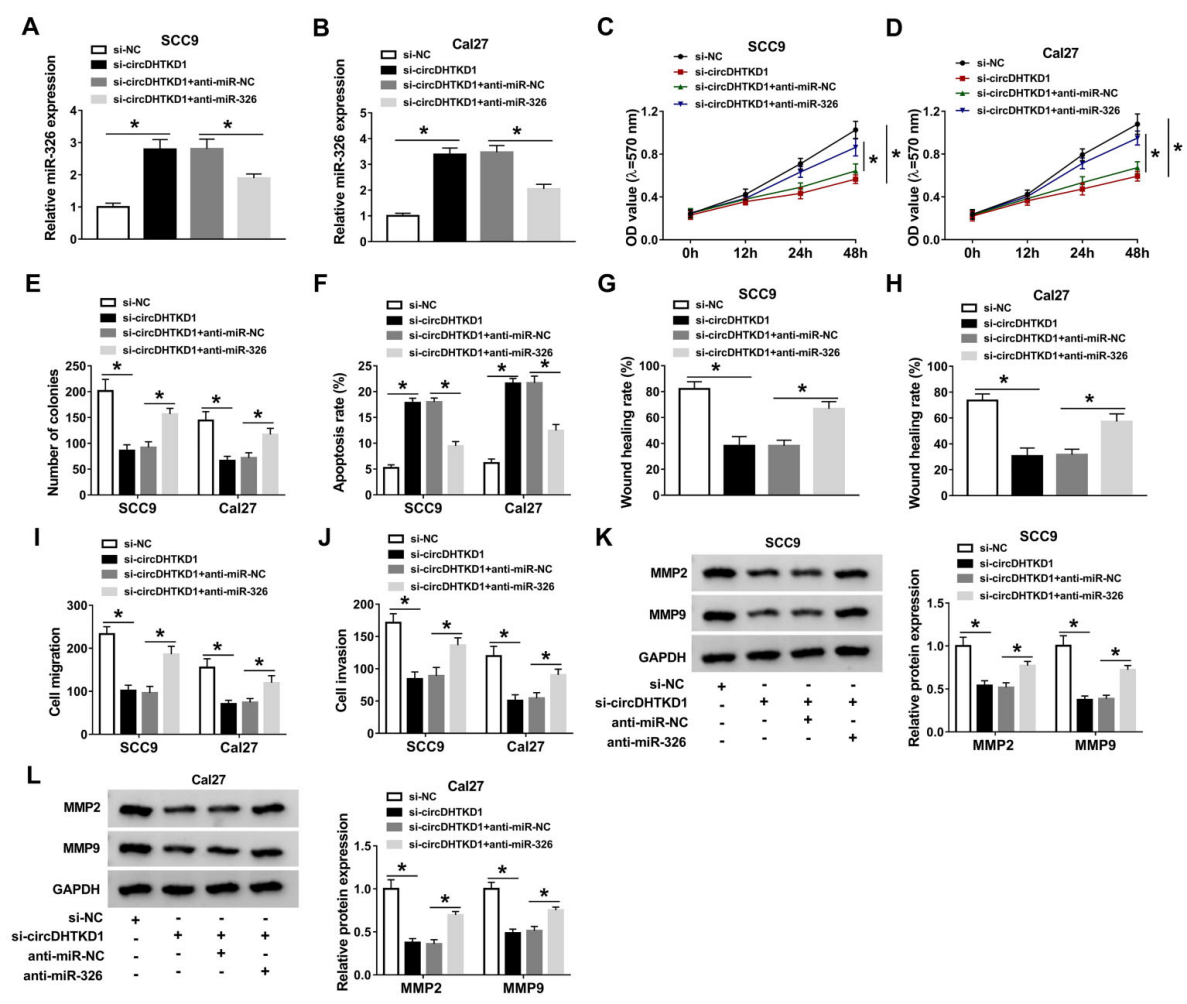
Inhibition of miR-326 partly restored the cancer-inhibitory function of downregulated circDHTKD1 in oral squamous cell carcinoma (OSCC) cells. **A** and **B**, The miR-326 level was measured through qRT-PCR after transfection of si-NC (negative control), si-circDHTKD1, si-circDHTKD1+anti-miR-NC, and si-circDHTKD1+anti-miR-326. Cell viability (**C** and **D**) and clonal capacity (**E**) were evaluated using MTT and colony formation assay. (**F**) The detection of cell apoptosis was completed via flow cytometry. Cell metastasis was assessed by wound healing and transwell assay for migration (**G**-**I**), as well as transwell assay and western blot for invasion (**J**-**L**). Three biological and three technical replicates were performed in each assay. Data are reported as means±SD. *P<0.05 (one-way ANOVA followed by Tukey's test).

Reversed experiment was performed through the transfection of si-NC, si-circDHTKD1, si-circDHTKD1+anti-miR-NC, and si-circDHTKD1+anti-miR-326. qRT-PCR demonstrated the large repressive efficiency of anti-miR-326 because of its significant neutralization for the si-circDHTKD1-induced miR-326 upregulation (Figure 4A and B). As the results of miR-326 inhibition, cell growth decline (Figure 4C-E) and apoptosis acceleration (Figure 4F) caused by silence of circDHTKD1 were recovered to a large extent. By conducting the metastasis-related assays (wound healing assay, transwell assay, and western blot), we found that the si-circDHTKD1-mediated inhibitory influences on migration (Figure 4G-I) and invasion (Figure 4J-L) were rescued via the introduction of miR-326 inhibitor. At least in part, the effects of circDHTKD1 knockdown on OSCC cells were related to miR-326 upregulation.

### circDHTKD1 regulated GAB1 expression via interacting with miR-326

**Figure 5:**
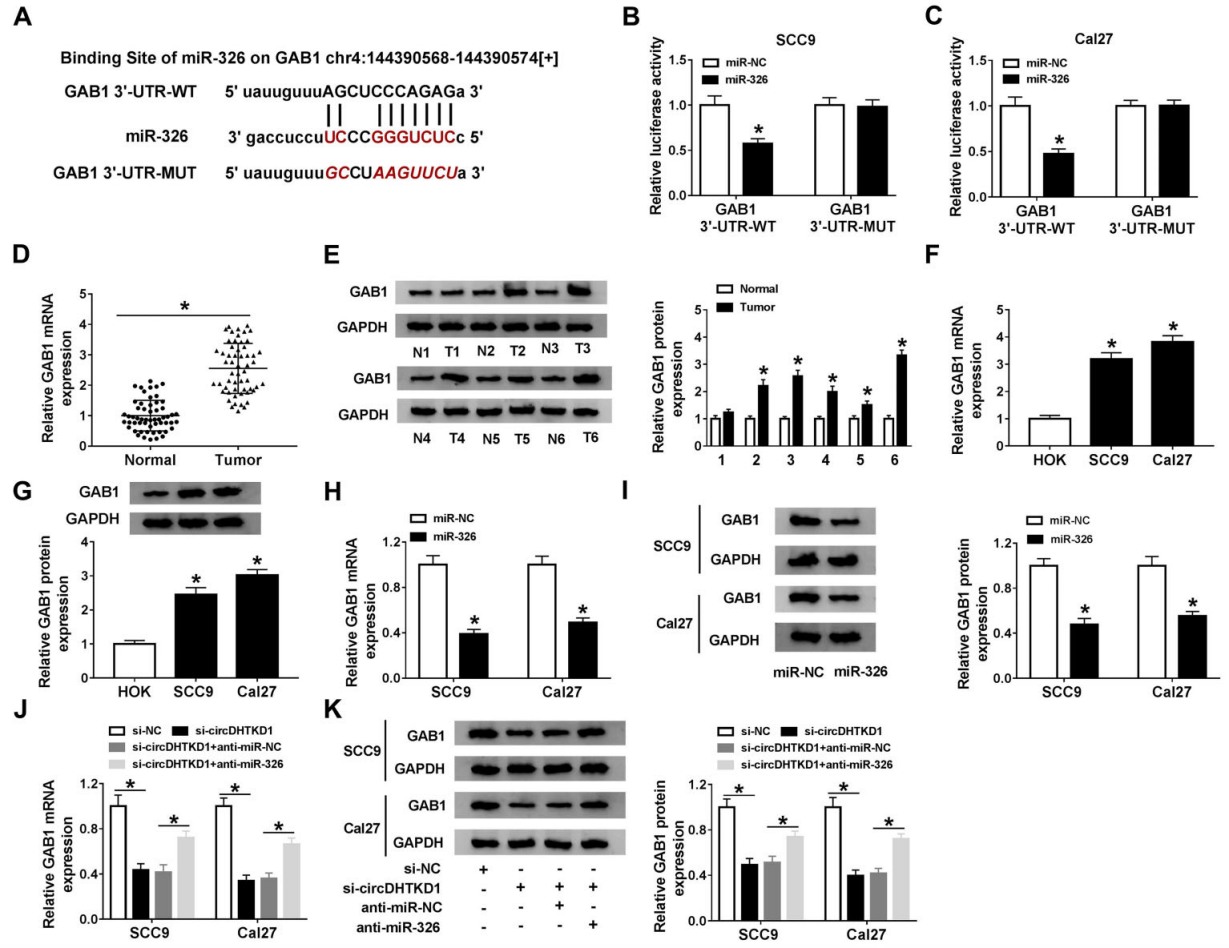
circDHTKD1 regulated GAB1 expression via interacting with miR-326. **A**, starBase was adopted to predict the binging region between miR-326 and GAB1 3′-UTR. **B** and **C**, Dual-luciferase reporter assay was used to determine whether miR-326 could combine with GAB1 3′-UTR in SCC9 and Cal27 cells. GAB1 mRNA and protein levels in OSCC tissues (**D** and **E**) and cells (**F** and **G**) were detected with qRT-PCR and western blot. qRT-PCR and western blot were used for assaying the mRNA and protein expression of GAB1 after transfection of miR-NC/miR-326 (**H** and **I**) and si-circDHTKD1, si-circDHTKD1+anti-miR-326, or the relative control groups (**J** and **K**). Three biological and three technical replicates were performed in each assay. Data are reported as means±SD. *P<0.05 (Student's *t*-test and one-way ANOVA followed by Tukey's test).

After the prediction of starBase, we noticed that the sequence of GAB1 3′-UTR contained the complemental sites for miR-326 (Figure 5A). In the dual-luciferase reporter assay, a decline was observed regarding the luciferase activity of GAB1 3′-UTR-WT but not of GAB1 3′-UTR-MUT vector by the overexpressed miR-326 (Figure 5B and C), revealing the combination of miR-326 and the 3′-UTR of GAB1. qRT-PCR and western blot showed the high mRNA and protein expression of GAB1 in OSCC tissues (Figure 5D and E) and cells (Figure 5F and G), compared with normal tissues and HOK cells. Additionally, GAB1 mRNA and protein levels were significantly reduced in miR-326-transfected SCC9 and Cal27 cells compared to miR-NC-transfected cells (Figure 5H and I). More interestingly, si-circDHTKD1 downregulated the mRNA and protein levels of GAB1, which were then reversed by miR-326 inhibitor (Figure 5J and K). Thus, GAB1 was regulated by the circDHTKD1-mediated miR-326.

### GAB1 downregulation was accountable for miR-326 as a tumor inhibitor in OSCC

**Figure 6:**
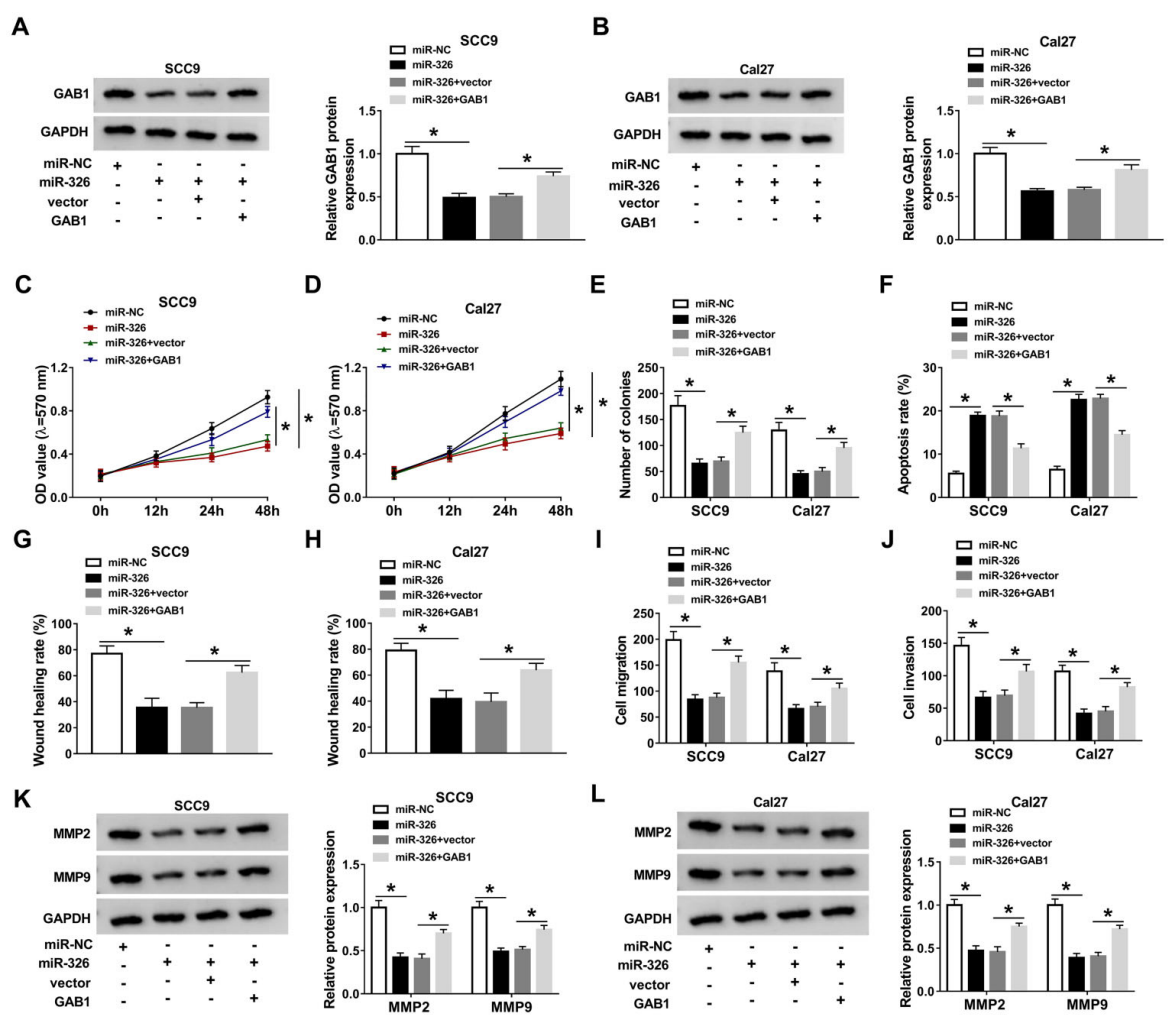
GAB1 downregulation was accountable for miR-326 as a tumor inhibitor in oral squamous cell carcinoma (OSCC). **A** and **B**, GAB1 protein analysis was conducted by western blot in SCC9 and Cal27 cells transfected with miR-NC (negative control), miR-326, miR-326+vector, and miR-326+GAB1. Cell growth was determined by cell viability via MTT (**C** and **D**) and clonal capacity via colony formation assay (**E**). **F**, Flow cytometry was used for assessing apoptotic cells. Wound healing assay, transwell assay, and western blot were used to assess cell migration (**G**-**I**) and invasion (**J**-**L**). Three biological and three technical replicates were performed in each assay. Data are reported as means±SD. *P<0.05 (one-way ANOVA followed by Tukey's test).

As for the function and mechanism analyses of miR-326 in OSCC, SCC9 and Cal27 cells were transfected with miR-NC, miR-326, miR-326+vector, and miR-326+GAB1. As shown in Figure 6A and B, GAB1 transfection eliminated the miR-326-induced inhibition of GAB1 protein expression. MTT and colony formation assays showed that miR-326 obstructed cell viability (Figure 6C and D) and colony formation capacity (Figure 6E), whereas these effects were antagonized by overexpressing GAB1. The apoptosis-promoting action in flow cytometry (Figure 6F) and migration inhibition in the wound healing assay (Figure 6G and H) by miR-326 were partially ameliorated after GAB1 was upregulated. In contrast with the miR-326 transfection group, migrated and invaded cells of the miR-326+GAB1 group were significantly increased in SCC9 and Cal27 cells (Figure 6I and J). The same counterbalance of GAB1 for miR-326 was found in the decrease of MMP2 and MMP9 proteins (Figure 6K and L). These findings revealed that miR-326 worked as a tumor inhibitor in OSCC by downregulating GAB1.

### circDHTKD1 promoted OSCC progression </emph>*in vivo* **by sponging miR-326 to regulate GAB1**

**Figure 7:**
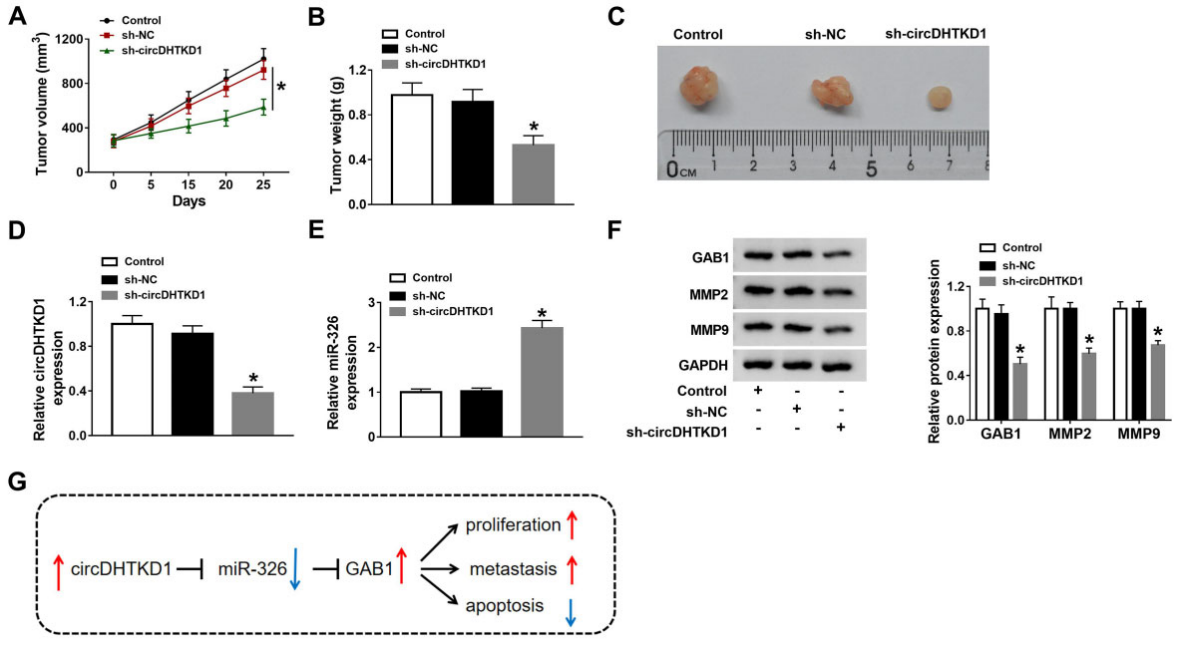
circDHTKD1 promoted oral squamous cell carcinoma (OSCC) progression *in vivo* by sponging miR-326 to regulate GAB1. **A**, Tumor volume was recorded after cell injection of control, sh-NC (negative control), and sh-circDHTKD1 groups every 5 days. Tumors were weighed after the excision from mice (**B**) and tumor images were photographed (**C**). **D** and **E**, The measurement of circDHTKD1 and miR-326 in tumor tissues was performed using qRT-PCR. **F**, Western blot was employed to determine the levels of GAB1, MMP2, and MMP9 proteins. **G**, A schematic figure of the current study. Three biological and three technical replicates were performed in expression detection. Data are reported as means±SD. *P<0.05 (one-way ANOVA followed by Tukey's test).

To further validate the potential of circDHTKD1 as a therapeutic biomarker in OSCC, we established the xenograft model through the injection of tumor cells in mice. After 25 days, tumor volume in the sh-circDHTKD1 group was smaller than that in control and sh-NC groups (Figure 7A). Tumor weight was also reduced after the knockdown of circDHTKD1 (Figure 7B and C). qRT-PCR suggested that circDHTKD1 expression was downregulated (Figure 7D) while miR-326 level was increased (Figure 7E) in the sh-circDHTKD1 group compared with control and sh-NC groups. Moreover, downregulation of circDHTKD1 evoked decreased protein expression of GAB1, MMP2, and MMP9 in tumors (Figure 7F). Thus, circDHTKD1 might be used as a therapeutic target for OSCC. Overall, circDHTKD1 could sponge miR-326 to upregulate GAB1 expression to promote tumor growth and metastasis in OSCC (Figure 7G).

## Discussion

circDHTKD1 is a novel circRNA with little exploration in cancer research. Fortunately, we have affirmed the carcinogenic action of circDHTKD1 in OSCC and its functional mechanism based on the miR-326/GAB1 axis in the current report, representing a comprehensive analysis of circDHTKD1 in OSCC.

Mounting evidence shows the abundant enrichment of circRNAs in human transcriptome (23). Recent studies have largely focused on the regulatory roles of circRNAs in various types of cancers. For example, circ_0016788 was revealed as a biological target for cancer progression and poor prognosis in hepatocellular carcinoma patients (24), and circENTPD7 enhanced the motility and proliferation of glioblastoma cells (25). We also found a high level of circDHTKD1 in human OSCC tissues and cells. In addition, circDHTKD1 showed circRNA properties with high stability and cytoplasmic localization in OSCC cells. As for the functions of circRNAs in squamous cell carcinoma, knockdown of circUBAP2 hampers the malignant tumor behaviors in esophageal squamous cell carcinoma (ESCC) cells (26); circ_081069 contributes to cellular proliferative and migratory abilities in tongue squamous cell carcinoma (TSCC) (27); and circ_0011946 accelerates cell growth and metastasis of OSCC (28). Our assays indicated that circDHTKD1 knockdown reduced cell growth and promoted cell apoptosis. Due to the high metastatic ability of OSCC, we further explored the effect of circDHTKD1 on cell metastasis. Through the conduction of the wound healing assay, transwell assay, and protein detection, we found that downregulation of circDHTKD1 triggered the obstruction of metastasis in OSCC cells. circDHTKD1 expression inhibition might be used as a targeted therapy for treating metastatic OSCC.

By using the online prediction software and Venn diagram analysis, we selected three miRNA targets for circDHTKD1 and finally affirmed that miR-326 was a definite target of circDHTKD1. circ_0001564 could regulate cell proliferation and apoptosis in osteosarcoma by playing the sponge role of miR-29c-3p (29), and circADAMTS13 reduced hepatocellular carcinoma cell proliferation by sponging miR-484 (30). Our reversed experiment exhibited that miR-326 downregulation mitigated the suppression of si-circDHTKD1 in the malignant progression of OSCC, suggesting that circDHTKD1 acted as an oncogenic factor in OSCC partly by sponging miR-326. However, the reversal of miR-326 inhibitor for the function of si-circDHTKD1 was not complete in OSCC cells. Thus, the regulation of circDHTKD1 in OSCC was also associated with other factors. Other miRNA sponges remain to be discovered for circDHTKD1 in OSCC.

GAB1 3′-UTR was found to be interplayed with miR-326, and miR-326 exerted a negative regulation on GAB1 expression in OSCC cells. GAB1 has been proven as a pro-cancer factor to promote proliferation in glioma (31), cell metastasis in breast cancer (32), and proliferation and invasion in non-small cell lung cancer (33). Herein, GAB1 also worked as an oncogene in OSCC to neutralize the miR-326-induced growth and metastasis inhibition, consistent with the previously published report of GAB1 in OSCC (17). Thus, OSCC progression was retarded by miR-326 that directly downregulated GAB1.

Through the sponge effect on miR-326, circDHTKD1 regulated GAB1 expression in a positive manner in OSCC cells. A good deal of circRNA/miRNA/mRNA regulatory networks have been unraveled in human cancers, like circAPLP2/miR-485-5p/FOXK1 in colorectal cancer (34) and circMYLK/miR-513a-5p/VEGFC in renal cell carcinoma (35). circDHTKD1/miR-326/GAB1 signal axis was first reported in OSCC in our current study. In the *in vivo* experiment, circDHTKD1 also facilitated tumorigenesis and invasion via modulating the miR-326/GAB1.

There are some limitations in the present study. Firstly, the reversed experiments of miR-326 and GAB1 for circDHTKD1 in the *in vivo* assay should be performed to better affirm the conclusion of circDHTKD1/miR-326/GAB1 axis in the regulation of OSCC. Secondly, miR-326 inhibitor did not reverse all those effects of si-circDHTKD1 on OSCC cells. The sponge function of circDHTKD1 on other miRNAs in OSCC progression remains to be explored. RNA sequencing after knockdown of circDHTKD1 may be a good manner to identify other potential miRNAs mediated by circDHTKD1. Thirdly, other downstream targets for miR-326 in OSCC should be explored. The different miRNA/mRNA axes will contribute to a better understanding of the functional mechanism of circDHTKD1 in OSCC.

In conclusion, circDHTKD1 increased the expression of GAB1 by sponging miR-326 in OSCC, inducing the promotion of tumor growth and metastasis *in vitro* and *in vivo*. This study developed a novel notion underplaying the OSCC carcinogenic mechanism at the molecular level by discovering the circDHTKD1/miR-326/GAB1 axis. circDHTKD1 may improve the diagnosis and treatment of OSCC as a potential biomarker.
